# Aspirin versus placebo for the treatment of venous leg ulcers—a phase II, pilot, randomised trial (AVURT)

**DOI:** 10.1186/s13063-019-3480-7

**Published:** 2019-07-26

**Authors:** Tilbrook Helen, Cook Liz, Clark Laura, Sbizzera Illary, Bland Martin, Buckley Hannah, Chetter Ian, Dumville Jo, Fenner Chris, Forsythe Rachael, Gabe Rhian, Harding Keith, Layton Alison, Lindsay Ellie, Mc Daid Catriona, Moffatt Christine, Rolfe Debbie, Stansby Gerard, Torgerson David, Vowden Peter, Williams Laurie, Hinchliffe Robert

**Affiliations:** 1 York Trials Unit, Department of Health Sciences, University of York, Heslington, York, YO10 5DD UK; 2 Department of Health Sciences, University of York, Heslington, York, YO10 5DD UK; 3 Cancer Division, Clinical Trials Research Unit, Leeds Institute of Clinical Trials Research, University of Leeds, Leeds, LS2 9JT UK; 4 Academic Vascular Surgical Unit, Hull Royal Infirmary, Anlaby Road, Hull, HU3 2JZ UK; 5 Division of Nursing, Midwifery and Social Work, School of Health Sciences, Faculty of Biology, Medicine and Health, University of Manchester, Manchester Academic Health Science Centre, Oxford Road, Manchester, M13 9PL UK; 6 Orthopaedic Department, West Middlesex Hospital, Twickenham Road, Isleworth, Middlesex, TW9 1UR UK; 7 Centre for Cardiovascular Science, University of Edinburgh, 49 Little France Crescent, Edinburgh, EH16 4SB UK; 8 Hull York Medical School & York Trials Unit, Department of Health Sciences, University of York, Heslington, York, YO10 5DD UK; 9 Wound Healing, Cardiff University, School of Medicine, Heath Park, Cardiff, CF14 4XN UK; 10 Harrogate and District NHS Foundation Trust, Lancaster Park Road, Harrogate, HG2 7SX UK; 11 (Lay representative). The Lindsay Leg Club Foundation, PO Box 689, Ipswich, IP1 9BN UK; 12 The University of Nottingham, School of Health Sciences, Derby Education Centre, Royal Derby Hospital, Uttoxeter Road, Derby, DE22 3DT UK; 13 Joint Research and Enterprise Office, St Georges University of London, Cranmer Terrace, London, SW17 0RE UK; 14 Freeman Hospital, Freeman Road, Newcastle upon Tyne, NE7 7DN UK; 15 Bradford Teaching Hospitals NHS Foundation Trust, Bradford Royal Infirmary, Duckworth Lane, Bradford, West Yorkshire BD9 6RJ UK; 16 Bristol Centre for Surgical Research, Bristol NIHR Biomedical Research Centre, University of Bristol, Canynge Hall, 39 Whatley Road, Bristol, BS8 2PS UK

**Keywords:** Trial, Pilot, Randomised, Aspirin, Placebo, Venous, Ulcer, Wound, Double-blind, Phase II

## Abstract

**Background:**

Venous leg ulcers (VLUs) can take many months to heal and 25% fail to heal. The main treatment for venous leg ulcers is compression therapy and few additional therapies exist. Two previous trials indicated that low-dose aspirin may improve healing time, but these trials were insufficiently robust.

**Methods:**

A multi-centred, pilot, phase II, randomised, double blind, parallel-group, placebo-controlled, efficacy trial (RCT) was conducted to determine: if aspirin improves VLU healing time; the safety of aspirin in this population; treatment compliance; and the feasibility of recruitment to a phase III trial. We recruited patients from secondary care who were aged ≥ 18 years, had a chronic VLU and not regularly taking aspirin. Participants were randomly assigned (1:1) to receive 300 mg of daily aspirin or placebo in addition to standard care, which consisted of multi component compression therapy aiming to deliver 40 mmHg at the ankle where possible. The randomisation list was stratified by ulcer size (≤ 5 cm^2^ or > 5 cm^2^). The primary endpoint was time to ulcer healing, which was defined as ‘complete epithelial healing in the absence of scab (eschar) with no dressing required’. Safety outcomes were assessed in all participants who received at least one dose of the study drug.

**Results:**

Twenty-seven patients were recruited from eight sites (target 100 patients). A short time-frame to recruit and a large number of patients failing to meet the eligibility criteria were the main barriers to recruitment. There was no evidence of a difference in time to healing of the reference ulcer following adjustment for log ulcer area and duration (hazard ratio 0.58, 95% confidence interval 0.18 to 1.85; *p* = 0.357). One expected serious adverse event related to aspirin was recorded. A number of options to improve recruitment were explored.

**Conclusions:**

There was no evidence that aspirin was effective in expediting the healing of chronic VLUs. However, the analysis was underpowered due to the low number of participants recruited. The trial design would require substantial amendment in order to progress to a phase III (effectiveness) trial.

**Trial registration:**

Clinicaltrials.gov, NCT02333123. Registered on 5 November 2014.

## Background

Chronic venous leg ulcers (VLUs) are open wounds which can be large and frequently become infected and leak exudates, leading to significant pain and reduction in quality of life [1, 2]. They are the result of an impaired venous return related to severe varicose veins, a history of deep vein thrombosis and trauma or failure of the calf muscle pump. Other contributing factors are obesity and immobility [3]. Prevalence of lower limb ulcers increases with age and is higher in women [4]. It is estimated that the prevalence of lower limb ulcers is around 1% of the population [4].

VLUs can take months to heal, have a tendency to become recurrent (estimated recurrence rates are between 18% and 23%) and approximately 25% fail to heal completely [1]. UK guidelines recommend the use of compression bandaging as first-line treatment for VLUs; its aim is to reduce venous hypertension, improve calf muscle function and create a wound environment that encourages healing while reducing maceration and excessive oedema and moisture. Randomised controlled trials (RCTs) have shown compression therapy to be effective [5], but healing can take many months (approximate median healing time is 12 weeks) [6]. Compression therapy is not always well tolerated by patients since it can be painful and inconvenient; bulky bandages may restrict ankle movement and cause difficulty in wearing shoes [7]. This may affect compliance to the treatment, which can consequently jeopardise its effectiveness. Additionally, this treatment can be expensive as, in addition to dressings and bandages, nurse time is required to change bandages, which can be required weekly or more frequently.

Some evidence from two small RCTs suggests that aspirin (300 mg/day) may improve VLU healing [8, 9]. Aspirin (acetylsalicylic acid) is inexpensive, readily available and generally safe to use. Aspirin is a cyclooxygenase inhibitor that irreversibly reduces prostaglandin and thromboxane A2 [10]. It is used widely to reduce cardiovascular events in those at high risk [11]. The exact mechanism by which aspirin may improve time to healing of VLUs is unclear but is potentially associated with both inhibition of platelet activation and reduction of inflammation [12, 13]. If aspirin was found to be effective for reducing healing time, with limited risk of treatment-related harm, it would present a significant reduction in resource use and improvements in health related quality of life. A Cochrane review has concluded that it is not possible to make definitive claims on the risks and benefits of oral aspirin on the recurrence and healing of VLUs due to the low quality and insufficient evidence from the two included RCTs [8, 9]; therefore, further high quality studies were recommended [12].

The AVURT trial (Aspirin for Venous leg Ulcers Randomised Trial) was conducted to address the primary question of whether the addition of 300 mg of daily aspirin to standard evidence-based therapies reduces VLU healing time. The pilot trial was also developed to assess the feasibility (especially in terms of participant recruitment and treatment compliance) and safety (in terms of aspirin-related adverse events) of conducting a larger pragmatic phase III study, powered to investigate the effectiveness and cost-effectiveness of aspirin for VLU healing.

Since the trial recruited fewer participants than expected, the “Results” section focuses mainly on feasibility aspects, such as adverse events, recurrence and compliance with study capsules. The results from the primary outcome analysis are also presented. The full results are reported in the HTA report, which is available on NIHR’s online Journal Library (https://www.journalslibrary.nihr.ac.uk/) [14].

## Methods

### Study design and participants

AVURT was a multi-centred, pilot, phase II, randomised, double-blind, parallel-group, placebo-controlled, efficacy trial. Ten recruiting centres participated, which included leg ulcer hospital outpatient clinics (*n* = 5), community leg ulcer clinics or community caseloads (*n* = 3), a wounds clinic within a university (*n* = 1) and a primary care leg ulcer clinic (*n* = 1). The full details of the inclusion and exclusion criteria for trial participants can be found in the study protocol, which has been published [15]. Briefly, the trial recruited adults (aged 18 years or over with no upper limit) with at least one chronic VLU, ulcer area > 1 cm^2^, ankle branchial pressure index (ABPI) ≥ 0.8 taken within the previous 3 months or, if the ABPI was incompressible, other forms of clinical assessment to exclude peripheral arterial disease (e.g. peripheral pulse examination, toe pressure, duplex ultrasound and clinical judgement). For patients with more than one ulcer, the largest ulcer was chosen as the reference ulcer for the purposes of the trial.

### Recruitment

**Fig. 1:**
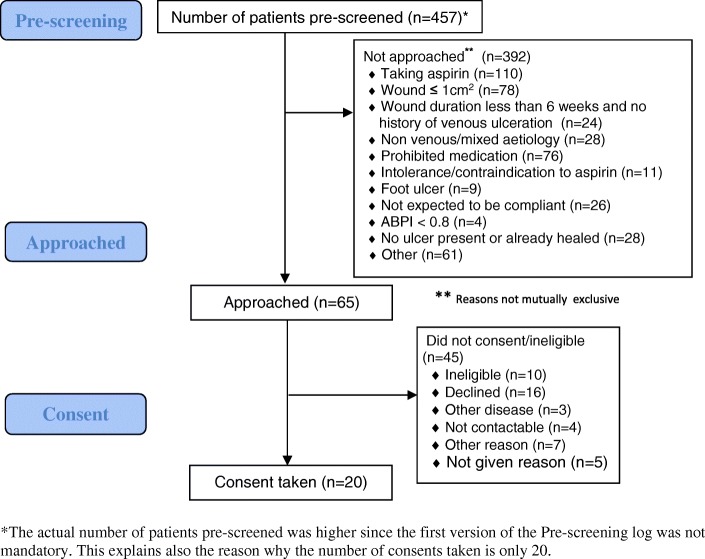
AVURT pre-screening study flow

Before approaching patients, study research nurses pre-screened on three criteria (concomitant aspirin, wound size and ulcer duration or history of venous ulceration) to determine those potentially eligible for the study. Two pre-screening logs were issued. The first, used during the first 4 months of recruitment, was non-mandatory as stipulated by the trial Sponsor. The second pre-screening, implemented in the fifth month of recruitment, was mandatory in accordance with a recommendation by the Data Monitoring Committee (DMC). Therefore, the number pre-screened as shown in the pre-screening study flow diagram (Fig. 1) is an under-representation of the number pre-screened as not all sites completed the non-mandatory log.

Potentially eligible patients visiting the clinics for a routine appointment were invited to take part in the trial by a designated health care professional, including research nurses. A patient information leaflet was provided and patients were given a minimum of 24 h to consider participation. A full eligibility check was conducted for patients who gave consent and an eligibility case report form (CRF) was completed by the health professional. Patients were informed during consent that their eligibility would be subject to confirmation by a doctor. Following review and confirmation of a patients’ eligibility by a doctor, the doctor signed off the prescription for the investigational medicinal product (IMP).

### Randomisation and masking

Participants were randomised on a 1:1 basis to receive either aspirin (300 mg) or placebo, in addition to standard care. The aspirin and placebo manufacturer, Sharp Clinical Services UK Limited, generated the randomisation schedule in advance. The schedule was stratified by ulcer area (≤ 5 cm^2^ or > 5 cm^2^) and mirrored top to bottom–bottom to top in order to facilitate participant allocation according to stratification. One randomisation list was provided to St George’s Hospital Research Pharmacy and a copy to the Senior Trial Statistician in the York Trials Unit (YTU). The Research Pharmacy was responsible for the randomisation, which was performed upon receipt of a valid prescription for a participant, and provided the 24-h code-breaking service in case emergency un-blinding was needed. Participants, investigators, research and treating nurses and other attending clinicians were unaware of the trial drug allocation throughout the trial.

### Intervention and comparator

Participants in the intervention group were allocated 300 mg of daily oral aspirin (single dose) for 24 weeks whereas participants in the control group were allocated daily oral placebo. Placebo capsules were identical in weight, colour and size to the aspirin capsules and contained the same filler.

Participants were offered an evidence-based standardised approach to the management of their leg ulcers in accordance with Scottish Intercollegiate Guideline Network (SIGN) guidance [16]. This consisted of multi-component compression therapy aiming to deliver 40 mmHg of pressure at the ankle, where possible. The type of dressing used was at the discretion of the healthcare professionals managing the participants.

### Procedure: baseline

After consent and confirmation by a doctor that the patient was eligible, but prior to randomisation, a nurse conducted a clinical assessment of the participant and wound. Baseline demographic and clinical details were recorded, including ulcer size, on a paper CRF. Medication that the patient was currently taking was recorded in a diary to be retained by the patient.

### Procedure: follow-up

Following randomisation, participants continued in the normal care pathway of weekly or fortnightly clinical assessments at community ulcer clinics, hospital outpatient clinics or home visits with no additional visits required for the study. Nurses completed follow-up paper CRFs at each visit to record assessments of healing outcomes, treatment concordance with IMP and compression bandaging, AEs or side effects, change of concomitant medication and resource use (e.g. number of visits, number of changes to dressings and changes to compression therapy). Digital photographs, or leg ulcer tracings, were also taken by the treating or research nurse at each visit.

Participants kept a medication diary to record any changes to their concomitant medication. The diary was reviewed by clinical staff at follow-up visits to monitor and record changes in medication. Changes in medication were subsequently reviewed by a doctor to ensure safety.

All participants were to be followed for 25 weeks.

### Procedure: healed ulcers

If the reference ulcer was assessed as healed during the follow-up period, a photograph was taken and the participant continued to take the aspirin or placebo for two further weeks. At this latter time point, they were re-assessed for healing (as per FDA guidelines on wound healing [17]). If the ulcer was confirmed as healed at the reassessment visit, the participant was advised to stop taking the IMP or placebo and the date of ulcer healing was recorded as the date the ulcer was first assessed as healed (i.e. 2 weeks earlier). Participants whose ulcer had healed no longer continued with regular follow-ups but were given a card with contact details of the research nurse at the site and the date of their 25-week follow-up. Participants were asked to contact the research nurse if a new ulcer occurred on the reference leg before the end of the study. If the ulcer was assessed as ‘not healed’ at the second assessment, the participant was advised to continue with the trial medication and continued to receive weekly or fortnightly follow-ups.

### Procedure: final follow-up

Final follow-up was 25 weeks post-randomisation. However, for participants whose leg ulcer was first assessed as healed in weeks 24–25, follow-up was extended to weeks 26–27, respectively, to allow a 2-week period of assessment to confirm whether the ulcer had healed. For all participants, no trial medication was taken after week 25.

For participants whose leg ulcer had healed before week 25, the research nurse or other nursing staff phoned participants in week 25 to ask if the reference ulcer had reoccurred and to check for adverse events. At the end of the study, participants were asked to return the bottle containing all the remaining capsules in order to assess compliance. St George’s Research Pharmacy conducted the pill count.

### Outcomes

The primary outcome was time to ulcer healing, which was defined as ‘complete epithelial healing in the absence of scab (eschar) with no dressing required’. Time to healing was measured in days from the date of randomisation until the first date that healing was recorded. Patients who did not heal or fully withdrew were censored at the end of the study or at their last visit, respectively. Secondary outcomes were: ulcer size (area) measured in cm^2^ using image analysis by SigmaScan (Systat Software Inc., San Jose, CA, USA) and/or wound tracings; recurrence of reference ulcer; adverse events; ulcer-related pain using a visual analogue scale; treatment compliance (patient self-assessment, capsule counting and nurse assessment of compression concordance); and resource use (i.e. number of wound consultations and types of dressing used).

Compliance with study capsules was assessed in two ways. The first was by analysing the answer given to the question in the CRF, which was completed at each visit: “How often has the participant taken their AVURT capsules (300mg Aspirin/placebo per day) this week?”. Numerical values were assigned to the responses available as follows: every day = 1, most days = 2, some days = 3, and not at all = 4. To calculate mean level of compliance with study capsules for each participant, the responses across all weeks following delivery of the capsules up to healing/trial exit were summed and divided by the number of visits attended. This was then categorised as fully compliant if the mean value was 1, partially compliant if the value was 2 or 3 and not at all compliant if the value was equal to 4. The second way of assessing capsule compliance was by the count of returned capsules at the end of the study. Each participant was given 190 capsules and by subtracting the number of returned pills it was possible to obtain an estimate of the number of capsules actually taken. The number of capsules which should have been taken was calculated starting from the date of first dose until 2 weeks after healing (for those who had healed) or the date of the last visit (for those who did not heal). From this the percentage of capsules each participant took (of those they should have taken) was calculated.

### Statistical analysis

The target sample size of 100 participants was considered to be sufficient to demonstrate whether there was evidence of an effect of aspirin to treat VLUs. The primary outcome was time to healing of the reference ulcer. Assuming a standard error for the hazard ratio (HR) of 0.105 following adjustment for log area and log duration of ulcer (as in a previous leg ulcer study with data on 448 participants, VenUS IV [18]) and applying this to the smaller sample size of 100 in this study implied that the standard error would be 0.22. A 95% confidence interval (CI) for the log hazard ratio would thus be log(HR) ± 0.435. Hence, if the hazard ratio for this study were the same as that suggested by previous studies (around 1.5 [8, 9]), the CI would be (0.97, 2.31) which just includes 1.00. It would be unlikely that if the HR is as suggested in the two previous studies that we would observe an overall HR below 1.00. Analyses were conducted using the principles of intention to treat. Time to healing was summarised using Kaplan-Meier curves and investigated using a stratified log-rank test. The primary analysis was a Cox proportional hazard regression adjusted for ulcer area (cm^2^) and duration at baseline, both logarithmically transformed; an unadjusted Cox PH regression analysis was also conducted. Adverse events (AEs) were reported overall and by trial arm in terms of number of participants with at least one event, total number of events and number of non-serious and serious AEs. Differences in total number of events by trial arm were compared using negative binomial regression adjusted for size and duration of ulcer (both log transformed). Ulcer recurrence was analysed descriptively reporting the number of patients who experienced a recurrence and the time to recurrence from healing. Compliance with study capsules (self-reported level of compliance and percentage of capsules taken) were presented descriptively.

## Results

**Fig. 2:**
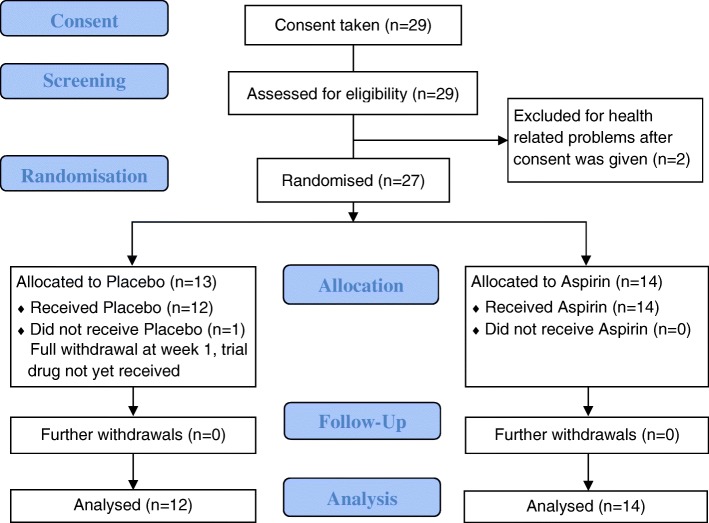
AVURT CONSORT diagram

The trial opened to recruitment on 23 June 2015 and closed to recruitment on 29 February 2016. Participant follow-up was completed on 18 August 2016. Consent was obtained from 29 patients but two were excluded due to health-related problems before randomisation. At the end of the recruitment period, only 27 patients were recruited and randomised (Fig. 2): 13 were assigned to the placebo group and 14 to the aspirin group. Figure 1 presents 20 patients who consented to the trial and who had pre-screening data. Figure 2 presents the total number of patients who gave consent (*n* = 29) and includes those for whom there was no pre-screening data.

One participant in the placebo group fully withdrew from the study without having taken any capsules and therefore no outcome data were collected. Four participants withdrew from treatment (two placebo, two aspirin) and either agreed and provided data until the end of planned follow-up or healed before withdrawal and thus all four provided primary outcome data. Baseline participant and ulcer-related characteristics are shown in Table 1. The average age of the 27 randomised participants was 62 years (SD 13) and two-thirds were male (*n* = 18). Median duration of reference ulcer was 15 months (range 2–234 months) and median size of ulcer was 17.1 cm^2^ (range 2.0–173.0 cm^2^). All participants were receiving compression therapy at baseline.

**Table 1:** Baseline data: participant and ulcer-related characteristics

	Placebo (*n* = 13)	Aspirin (*n* = 14)	Overall (*n* = 27)
Participant characteristic			
Age			
Mean (SD)	62.1 (15.2)	62.7 (11.6)	62.4 (13.2)
Median (min, max)	66.6 (38.9, 80.8)	59.2 (47.9, 78.9)	62.0 (38.9, 80.8)
Gender, n (%)			
Male	7 (53.9)	11 (78.6)	18 (66.7)
Female	6 (46.2)	3 (21.4)	9 (33.3)
BMI			
Mean (SD)	32.1 (8.6)	36.6 (15.0)	34.4 (12.3)
Median (min, max)	28.4 (19.9, 44.1)	31.6 (20.9, 70.2)	31.5 (19.9, 70.2)
Ulcer related characteristics			
Size of ulcer (cm^2^)			
Mean (SD)	40.7 (55.1)	43.1 (47.6)	42.0 (50.3)
Median (min, max)	16.0 (2.0, 173.0)	31.3 (3.8, 155.0)	17.1 (2.0, 173.0)
≤ 5 cm^2^, n (%)	3 (23.1)	3 (21.4)	6 (22.2)
> 5 cm^2^, n (%)	10 (76.9)	11 (78.6)	21 (77.8)
Time since first ulcer (months)			
Mean (SD)	112.5 (78.5)	86.4 (86.9)	99.0 (82.4)
Median (min, max)	101.0 (11.0, 240.0)	48 (2.2, 240.0)	72.0 (2.2, 240.0)
Reference ulcer duration (months)			
Mean (SD)	58.6 (73.3)	32.2 (52.0)	44.9 (63.3)
Median (min, max)	13.0 (4.0, 234.0)	16.5 (1.8, 192.0)	15.0 (1.8, 234.0)
ABPI			
Mean (SD)	1.1 (0.2)	1.0 (0.1)	1.0 (0.2)
Median (min, max)	1.0 (0.8, 1.5)	1.0 (0.9, 1.3)	1.0 (0.8, 1.5)

A total of 13 participants were recorded as healed during the course of the study: seven (58.3%) participants in the placebo group and six (42.9%) in the aspirin group. All the reference ulcers reported to be healed were confirmed as such approximately 2 weeks later. It was not possible to estimate the median time to healing and/or corresponding 95% CIs where less than 50% of considered participants healed during the follow-up period of the study (e.g. in the aspirin group). Therefore, the 25th percentile of time to healing was also estimated (Table 2).

**Table 2:** Healing of the reference ulcer (unadjusted analysis)

	Placebo (*n* = 12)^*^	Aspirin (*n* = 14)	Overall (*n* = 26)
Number healing (n, %)	7/12 (58.3)	6/14 (42.9)	13/26 (50.0)
Kaplan-Meier estimate of median time to healing (days) (95% CI)	98 (21, NE)	NE (84, NE)	147 (97, NE)
Kaplan-Meier estimate of 25th percentile time to healing (days) (95% CI)	36 (20, 97)	111 (69, NE)	84 (21, 111)

*NE* not possible to estimate

*One participant was lost to follow-up immediately after randomisation and provided no outcome data

**Fig. 3:**
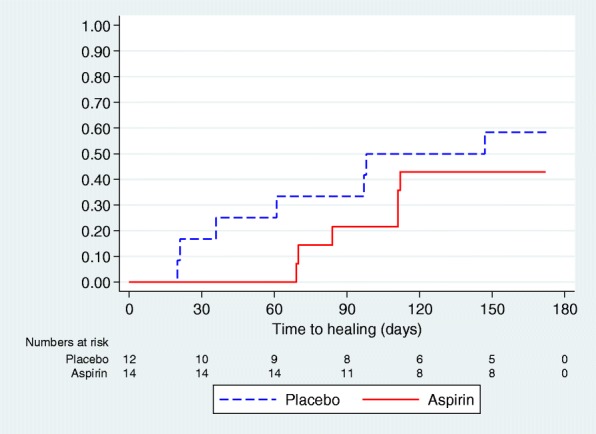
Kaplan-Meier plot of time to ulcer healing by trial arm (unadjusted)

Figure 3 shows the Kaplan-Meier plot of proportion of reference ulcers healed over time by trial arm. The log-rank test investigating the difference between the survival curves showed no statistically significant difference (*p* = 0.31). Hazard ratios (HRs), corresponding 95% CIs and *p* values for the model covariates are reported in Table 3.

**Table 3:** Healing of the reference ulcer (log-rank test, unadjusted and adjusted analysis)

	Test statistic	*P* value
Log-rank test	1.02 (1df)	0.312
Unadjusted Cox regression		
Parameter	HR (95% CI)	*P* value
Aspirin vs placebo (allocation)	0.58 (0.19, 1.72)	0.322
Adjusted Cox regression		
Parameter	HR (95% CI)	*P* value
Aspirin vs placebo (allocation)	0.58 (0.18, 1.85)	0.357
Area (log transformed)	0.42 (0.22, 0.81)	0.009
Duration (log transformed)	0.61 (0.34, 1.08)	0.089

The placebo group tended to heal more rapidly but this difference is not statistically significant (adjusted HR 0.58 [95% CI 0.18–1.85]). Overall, these data do not provide evidence of a difference in time to healing with the addition of aspirin to usual care.

Six of the 26 (23.1%) participants followed up had no reported AEs (three placebo, three aspirin) and the remaining 20 had at least one AE (nine placebo, 11 aspirin). In total 88 non-serious AEs (36 placebo and 52 aspirin) and one serious AE (“blood transfusion for low Hb” (haemoglobin); classified as expected and probably related to aspirin) were registered. There was no evidence from an adjusted negative binomial regression that participants receiving aspirin were more likely to suffer an AE than those receiving placebo (incidence rate ratio 1.31 [95% CI 0.51–3.41], *p* = 0.58). Among the 13 participants who healed, one recurrence was reported in each of the treatment groups. The time between ulcer healing and recurrence was 158 days for the participant in the placebo group and 126 days for the participant in the aspirin group.

According to self-assessment of compliance, eight of the 12 participants (66.7%) in the placebo group were deemed fully compliant with study capsules while four (33.3%) were partially compliant. In the aspirin group, 11 of the 14 participants (78.6%) were deemed fully compliant and three (21.4%) were partially compliant. Among partially compliant participants, two in the placebo group and two in the aspirin group were fully compliant for at least 88% of their visits. Reasons for not being fully compliant included illness, forgetting to take the capsule and experiencing an adverse effect. The other three participants (two placebo, one aspirin) were deemed to be fully compliant for 54% or less of their visits. Two of these participants (one aspirin, one placebo) were withdrawn from treatment at week 8 and week 14, respectively, while one participant (placebo) tended to forget to take the capsule. According to the count of returned capsules, ten participants in the placebo arm (83.4%) and ten in the aspirin arm (71.5%) took at least 90% of the study capsules they should have taken.

### Barriers to patient recruitment

#### Several factors contributed to the low recruitment rate

The key factor was that, during recruitment, sites found fewer than estimated eligible patients. The main reasons for ineligibility reported in the pre-screening forms were the current consumption of aspirin or other prohibited medication, the size of the ulcer (too small) and the type of ulcer (see flow diagram in Fig. 1). Feedback from sites suggested that many leg ulcer patients were treated in primary care settings and in specialist clinics; patients being seen in the secondary care recruiting sites taking part in this study were more likely to be older and therefore already having comorbidities requiring the consumption of aspirin.

Other factors were a 2-month suspension in opening to recruitment which was due to a delay in the release of the IMP just prior to recruitment. Once the IMP had been released, sites were slower than anticipated to open to recruitment due to limited staff availability over the summer months and at three sites key staff were on long-term leave or were waiting for new staff to be in post.

#### Recruitment strategies considered

In order to improve recruitment, several strategies were considered. The trial had 6 months initially to recruit (this was later extended to 8 months) and so it was necessary to adopt strategies that could be implemented relatively quickly.

##### Change to eligibility criteria

The only acceptable modification to the eligibility criteria was to include a wound area of less than 1 cm^2^. This would bring the trial in line with the concurrent leg ulcer trial in New Zealand [19], which included small wounds. However, we chose not to include these in the initial design stage of the trial as these ulcers tend to heal rapidly. To include patients already taking aspirin would have resulted in a significant redesign of the trial, e.g. patients randomised to the intervention would receive a top-up dose of aspirin to 300 mg or patients randomised to the control group would be asked to stop taking aspirin. However, this was not considered feasible for ethical and practical reasons; participants would be unblinded to treatment and a lower dose IMP would have been required.

##### Recruitment from primary care

The trial’s DMC supported a potential new strategy for the trial of recruiting from primary care. One option was to use GP practices as recruitment and treatment centres. However, time and budget constraints related to implementing this strategy meant that this was not a viable option. Another option was to use GP practices as patient identification centres (PICs) who would refer patients to the recruiting sites; however, preliminary searches of GP practice databases indicated that this too was not a viable option; 14 GP practices in England identified a total of seven potential patients. In addition a couple of recruiting sites had waiting lists for their wound service and therefore would have been unable to take additional referrals from primary care.

##### Others

Other recruitment strategies considered were media advertising (on the radio for example), posters for patients in recruitment sites and allowing more telephone follow-up so that participants who did not visit clinics as regularly as once a week or fortnightly could be recruited.

##### Strategies implemented

Flyers to sites to remind clinic staff to recruit to the trial and three electronic newsletters to update on recruitment to the trial were implemented since these were relatively cheap to produce and did not require ethics approval. Sites were also reminded to identify potential participants before site opening so that they could be approached as soon as they were given the green light to recruit.

## Discussion

This phase II pilot trial recruited only 27% of its target sample size but has important findings for informing the design of future clinical trials of IMPs for this patient population. There were a number of challenges to patient recruitment, including a short recruitment window (initially 6 months, extended to 8 months), a delay in the release of the IMP, staff shortages and absences at some sites, and many patients not meeting the trial’s eligibility criteria; for one recruiting site, the usual care pathway for the majority of VLU patients was for follow-up treatments to be delivered in the community by nurses and therefore the site was only able to recruit the few patients that had follow-up at the site.

There was no statistically significant difference between the two groups with respect to the proportion of ulcers that had healed. Ulcers tended to heal more rapidly in the placebo group but this was not statistically significant. Due to the low number of participants in the trial (*n* = 27), the efficacy of aspirin cannot be confirmed and related data should be interpreted with caution. However, recent evidence from another study also found that 300 mg of aspirin is not effective for time to healing of VLUs and, consistent with our study, although there was no statistically significant significant difference with respect to healing, the direction of the reported effect favoured the placebo group [20].

All the registered AEs were non-serious, with the exception of one serious event. There was no evidence of a difference in the number of expected AEs in the two trial arms and aspirin appeared to be generally well tolerated. Both measures of compliance with trial medication (self-report and pill count) showed good levels of compliance. Overall nearly three-quarters of participants were fully compliant and one-quarter partially compliant. Reasons for not being fully compliant included forgetting to take the medication, illness and experiencing an AE.

Although sites initially indicated that they could recruit, it became apparent once recruitment was open that a large proportion of patients seen did not meet the trial’s criteria. In order to improve recruitment, we explored a range of options, including recruitment from primary care, which involved a limited database search of records held in primary care. Very few patients were identified (*n* = 7 patients in 14 GP practices) but there were limitations associated with conducting the search. Further investigation from primary care was not explored due to these preliminary findings indicating that recruitment from primary care might not be effective and due to consideration of the time and budget constraints on the project. However, future studies should consider conducting an audit to identify the number of potential participants.

During the registration of AVURT, we identified two full scale randomised controlled trials being conducted outside the UK investigating aspirin for venous leg ulcers [19, 21]. The Aspirin4VLU trial [19], conducted in New Zealand, investigated low dose aspirin (150 mg) and had similar inclusion and exclusion criteria, but in contrast had no minimum ulcer size and recruited from a district nursing setting. The trial was able to recruit 251 participants [20] (ClinicalTrials.gov, NCT02158806), which suggests that clinical setting and ulcer size may have been important factors. The ASPiVLU trial [21] in Australia is currently ongoing.

### Limitations

It was estimated that 100 patients were sufficient to demonstrate whether there was evidence of an effect of aspirin to treat VLUs. As this trial recruited only 27 patients we were unable to determine the effectiveness of aspirin for this condition.

### Generalisability

The majority of the recruiting sites were based in secondary care. The participants recruited tended to be aged in their early 60s and have a high BMI score. More men (*n* = 67%) than women were recruited. Around a quarter of patients were excluded because they were already taking aspirin.

## Conclusions

AVURT was a phase II randomised pilot trial of aspirin versus placebo for the treatment of patients with chronic VLUs which sought to identify the efficacy of 300 mg of oral aspirin, feasibility of recruitment, compliance with treatment and the safety of high dose aspirin in the VLU patient population. The placebo group tended to heal more rapidly but this was not statistically significant. Aspirin appeared to be well tolerated and levels of compliance were good.

Due to the low number of participants, the efficacy of aspirin cannot be confirmed and any data should be interpreted with caution. The trial was not able to recruit the target number of patients despite a short unfunded extension to the trial’s recruitment phase and therefore it was not feasible to proceed to a larger phase III (effectiveness) trial without significantly changing the trial design, including eligibility criteria. Key barriers to recruitment were many patients not meeting the eligibility criteria and a short recruitment window.

It is recommended that prior to conducting a clinical trial of an IMP in this patient population, a thorough audit is carried out to determine the number of potential patients available for recruitment from secondary or primary care. The audit should consider the number of patients already taking the drug being evaluated and prohibited medication. In order for this pilot trial to progress to a full scale trial and to successfully recruit from secondary care without any amendments to the eligibility criteria, it would need many recruiting centres and require a long recruitment phase.

The full report for the study is available on NIHR’s online Journals Library (https://www.journalslibrary.nihr.ac.uk/hta/hta22550/#/full-report).

## Data Availability

All available non-identifiable data can be obtained for research purposes from the York Trials Unit via the corresponding author.

## Abbreviations

ABPI: Ankle brachial pressure index
AE: Adverse event
CI: Confidence interval
CTIMP: Clinical trial of an investigational medicinal product
DMC: Data Monitoring Committee
FDA: United States Food and Drug Agency
HR: Hazard ratio
HTA: Health technology assessment
IMP: Investigational medicinal product
MHRA: Medicines and Healthcare Products Regulatory Agency
NIHR: National Institute for Health Research
NRES: National Research Ethics Service
REC: Research Ethics Committee
TSC: Trial Steering Committee
